# Changes in alpha-amylase activity, concentration and isoforms in pigs after an experimental acute stress model: an exploratory study

**DOI:** 10.1186/s12917-018-1581-2

**Published:** 2018-08-29

**Authors:** María Dolores Contreras-Aguilar, Damián Escribano, Silvia Martínez-Subiela, Silvia Martínez-Miró, Jose Joaquín Cerón, Fernando Tecles

**Affiliations:** 1 Interdisciplinary Laboratory of Clinical Analysis (Interlab-UMU), Veterinary School, Campus of Excellence Mare Nostrum, University of Murcia, 30100, Espinardo, Murcia, Spain; 2 Department of Food and Animal Science, School of Veterinary Medicine, Universitat Autònoma de Barcelona, 08193, Bellaterra, Barcelona, Spain; 3 Department of Animal Production, Veterinary school, Campus of Excellence Mare Nostrum, Murcia, Spain

**Keywords:** Biomarkers, Isoforms, Salivary alpha-amylase, Stress, Swine

## Abstract

**Background:**

Salivary alpha-amylase (sAA) is considered a non-invasive biomarker of acute stress that can be evaluated by changes in activity and concentration, and also by changes in its isoforms, although this last way of evaluation has never been used in veterinary medicine. This research evaluated the changes of sAA by three different ways in which sAA can be evaluated in an experimental acute stress model in six pigs based in a technique of temporarily restraining. These ways of evaluation were 1) activity by a spectrophotometric assay, 2) concentration by a fluorometric assay, and 3) isoforms of the enzyme by a Western blot.

**Results:**

Although salivary cortisol significantly increased due to the stimulus of stress and all the pigs manifested signs of stress by high-pitched vocalization, sAA activity showed an increase of different degree in the six pigs after the stress stimulus, while sAA concentration showed decreases in four of the six pigs. sAA activity did not correlate with sAA concentration or salivary cortisol, and a low correlation was observed between sAA concentration and salivary cortisol (*r* = 0.48, *p* = 0.003). The inter-individual variability was higher in sAA activity than in sAA concentration and salivary cortisol. Finally, three possible isoforms of sAA at 154–160 kDa, 65–66 kDa and 59–60 kDa were observed that showed different dynamics after the stress induction.

**Conclusions:**

Although this pilot study’s results should be taken with caution due to the low sample size, it reveals a different behavior between sAA activity and concentration in pig after an acute stressful stimulus leading to evident external signs of stress by high-pitched vocalization, and opens a new field for the evaluation of possible selected isoforms of sAA as potential biomarkers of stress.

**Electronic supplementary material:**

The online version of this article (10.1186/s12917-018-1581-2) contains supplementary material, which is available to authorized users.

## Background

Nowadays there is an interest in improving the stress evaluation in highly productive indoor-housed pigs since stress can induce behavioral, physiological and immunological alterations leading to a reduction of animal welfare and health [1]. The stress can be evaluated by direct behavioral observations, for example using behavior scoring systems [2–4] or by recording high-pitched vocalizations [5, 6]. However, it can be also objectively assessed by biomarkers that can reflect the pathophysiological responses to stress [7]. Currently there is a growing interest in the use of saliva as a sample for measurement of stress biomarkers [8–11] because saliva is easily obtained and non-invasively compared to blood [12]. Salivary alpha-amylase (sAA) has been proposed as a biomarker for assessing acute psychological stress in humans due to sympathetic activation [13, 14]. In addition, sAA has been also measured in pig as a sympathetic nervous system (SNS) marker [15], whereas cortisol has been measured to assess activation of hypothalamic–pituitary–adrenocortical (HPA) axis [7, 16]. In addition to pigs, sAA has been studied in other animal species such as bonobos and other simians [17], horses [10], sheep [18] and dogs [19].

sAA can be evaluated by its enzymatic activity, although a high inter-individual variability in response to stress has been reported in pigs [15], as well in humans [13] and horses [10]. Quantification is another way to evaluate sAA, showing a lower inter-individual variability, as described in horses [10] and humans [20]. Lastly, sAA isoforms and its possible changes after a sAA stimulation have been evaluated in humans [21, 22]. Despite the interest and use of this enzyme, to the authors` knowledge there are no studies comparing the different ways in which the sAA response against a stressor could be assessed: activity, concentration and changes in isoforms.

The aim of this research was to evaluate the individual changes of sAA in an experimental acute stress model in pig, consisting of a nasal snare immobilization [23], by the three different ways in which sAA can be evaluated: (1) measurement of activity with a spectrophotometric assay, (2) measurement of concentration with a time-resolved immunofluorometric assay (TR-IFMA), and (3) the study of the possible changes in isoforms by western blot (WB). In addition, the sAA results were compared to those obtained with salivary cortisol.

## Methods

### Animals and experimental acute stress model

For this study, male crossbreed pigs (Duroc × (Landrace × Large White) in the mid-fattening period (mean age = 104.8 ± 10.0 days, mean body weight = 78.3 ± 6.3 Kg) from the high sanitary/health-status farm unit of the University of Murcia (Spain) were used. All animals were subjected to a clinical examination prior to the study and no clinical signs of disease were detected. The pigs had access to a nutritionally balanced diet (commercial dry diets based in a corn-soybean meal with 15.5% of crude protein -CP-; 0.79% Ileal digestible Lys, %; and 13.5 MJ of metabolizable energy per kg -ME/kg-) and water ad libitum (from nipple drinkers) under general commercial housing and husbandry conditions conforming to the European Union Guidelines (Directive 2010/63/EU1). Each pen had an area of 1.139m^2^ per animal, being in concordance to the legislation (Council Directive 2001/88/CE). The temperature in the pens was kept between a minimum of 18.0 °C and a maximum of 19.5 °C. The Murcia University Ethics Committee with the number CEEA 288/2017 approved this study.

The stressful situation consisted of a technique of temporarily restraining pigs commonly used in veterinary practice which has been demonstrated to produce a high stress to pigs [7, 23]. Six pigs were subjected to an immobilization for at least 1 min with a nasal snare or loop following the procedure described in the literature [15, 23]. Salivary samples were taken the day before the experiment took place, which was considered as basal time (Tb). On the other hand, the same day of the experiment samples were taken 5 min before restrain (T-5), for the first 30 s while the nasal snare immobilization was performed (T + 0A), for the second 30 s during immobilization (T + 0B), just after immobilization (T + 0C) and 15 min later (T + 15). The saliva collection at each time lasted 1 min, except for T + 0A and T + 0B. The sampling times were selected to evaluate the possible short-term changes in sAA. The observer responsible for sampling individually recorded if high-pitched vocalizations were present or absent during the restraint period. All animal were sampled between 9:00 to 12:00 a.m. to avoid circadian influences [24, 25], by the same person, when animals were in repose, avoiding the race and from pigs housed at different rooms to avoid suggestion; in order to homogenize the experimental conditions for all animals.

### Salivary sampling

Saliva samples were collected by introducing a small sponge around the mouth and it was placed in a collection device (Salivette, Sarstedt, Aktiengesellschaft & Co, Nümbrecht, Germany), as reported before [23]. Samples were then stored in ice until arrival at the processing laboratory, where devices were centrifuged at *3.000*×*g* for 10 min at 4 °C. Saliva was transferred into 1.5 mL tubes and stored at − 80 °C until analysis.

### Alpha-amylase enzymatic assay

sAA activity was measured in an automatic analyzer (Olympus UA600, Olympus Diagnostica GmbH) by a colorimetric commercial kit (Alpha-Amylase, Beckman Coulter Inc.) following the International Medicine (IFCC) method [26]. This spectrophotometric assay uses 4,6-ethylidene(G7)-p-nitrophenol(G1)-alpha-D- maltoheptaoside (G7PNP) as a substrate of the enzyme. The intermediate product of substrate hydrolysis reacts with a-glucosidase, giving p-nitrophenol as the final product of the reaction. The rate of p-nitrophenol formation was directly proportional to the alpha-amylase activity of the sample and was determined by measuring the absorbance at 405 nm. This kit was previously validated for pigs by Fuentes et al. [15], with a intra- and inter-assay coefficients of variation (CV) lower than 10%, and a limit of detection of 11.65 U/L.

### Time-resolved immunofluorometric assay

sAA concentration was measured by a time-resolved immunofluorometric assay (TR-IFMA). The assay consisted of a non-competitive indirect sandwich method based on anti-human sAA polyclonal antibody biotin-labeled as a capture reagent and the anti-human-sAA polyclonal antibody Eu^3+^−chelates labeled as a detector. Streptavidin-coated plates (Streptavidin Microtitration Strips, DELFIA, PerkinElmer, Turku, Finland) were used for the development of this assay. The anti-human sAA polyclonal antibody used was produced by the authors according to standard protocols [27] and using as immunogen human sAA purified in the researchers’ laboratory according to the procedures of Peng et al. [28].

The fluorometric assay was validated prior to use for sample measurement. Three saliva samples with a low, medium and high sAA mean concentration (10.7, 30.0 and 65.2 ng/mL, respectively) were measured five times in a single run and in five different days for the determination of intra- and inter-assay precision, respectively. The mean CV was 6.8% ± 0.8% for intra-assay, and 7.4% ± 2.3% for inter-assay. Linearity under dilution of two saliva samples yielded Pearson correlation coefficients of *r* = 0.989 ± 0.006 (mean ± SD). The limit of detection and the lower limit of quantification calculated were 1.4 ng/mL and 18.14 ng/mL, respectively.

### Western blot

WB was performed in the saliva collected at Tb, T + 0A, T + 0B and T + 0C of three pigs that showed different sAA activity response: pig 1 that showed the highest difference of sAA activity levels, pig 3 that showed intermediate differences and pig 5 that showed the lowest difference in sAA activity along the times. Saliva from pigs diluted to obtain an amount of 1 ng of sAA and 10 μg of purified human sAA (ab 77875, Abcam, Cambridge, UK) were used for this approach. Proteins were separated in mini one-dimensional sodium dodecyl sulphate polyacrylamide gels (SDS-PAGE) containing 0.1% (*w*/*v*), with a separating gel prepared in 12% (w/v) and a stacker gel prepared in 4% (w/v) according to the methodology described by Laemmli [29]. Then, the proteins in SDS-PAGE were transferred to nitrocellulose membranes (Bio-Rad Laboratories Inc., Hercules, CA, USA). The same rabbit polyclonal antibody against human sAA used in the TR-IFMA (1:8000 dilution) was employed as primary antibody, while anti-rabbit IgG horseradish peroxidase (HRP)-conjugated goat polyclonal antibody (ab 6721, Abcam, Cambrigde, UK) (1:12000) was used as secondary antibody, and signal was detected using Pierce ECL2 kit (Pierce, Thermo Fisher Scientific, USA) and ImageQuant™ scanner (GE Healthcare, Uppsala, Sweden). The average intensity of each band, defined as the volume of the band feature divided by its area, was analyzed by using ImageQuant™ TL 8.1 (GE Healthcare, Uppsala, Sweden).

### Cortisol measurement

Salivary cortisol was evaluated also for comparative purposes [30] with an automated chemiluminescent immunoassay (Immulite 1000 cortisol, Siemens Medical Solutions Diagnostics) validated for pigs’ saliva [8]. The intra- and inter-assay CV was lower than 16%, and a limit of detection of 0.016 μg/dL.

### Statistical methods

Arithmetic means, standard deviations (SD), CV and linear regression analyses were calculated using routine descriptive statistical procedures by spreadsheet (Excel 2000, Microsoft Corporation, Redmond, Washington, USA). To determine if sAA and salivary cortisol values obtained at different times in the stressful situation were statistically different, a Friedman test and a Dunn’s multiple comparisons test were used. A Spearman correlation test was performed to compare sAA activity, sAA concentration and salivary cortisol results. The inter-individual variability of the sAA values (activity and concentration) and salivary cortisol in each time were expressed as the CV value, calculated as the SD divided by the mean of the values of the different individuals and then multiplied by 100. These statistical analyses were calculated using Graph Pad Prism 6 (GraphPad Software, San Diego, CA, USA).

## Results

### Activity and concentration of alpha-amylase in saliva

**Fig. 1:**
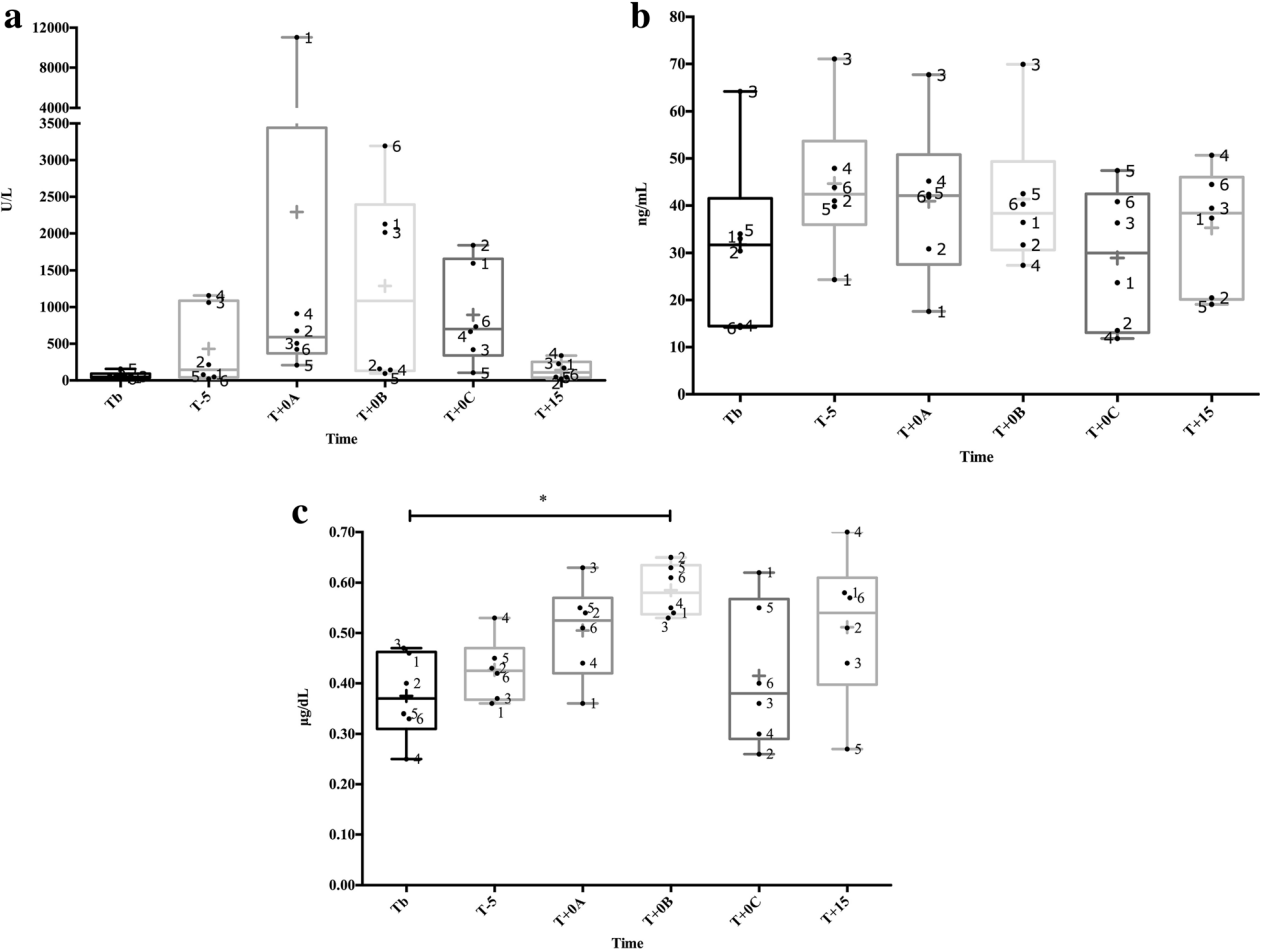
Salivary alpha-amylase (sAA) activity (**a**), concentration (**b**) and salivary cortisol (**c**) during a nasal snare immobilization for 1 min from six pigs. The times were the day before at basal time (Tb), 5 min before (T-5), the firsts 30 s while the nasal snare immobilization was being performed (T + 0A), the seconds 30 s while the nasal snare immobilization was being performed (T + 0B), just after the immobilization (T + 0C) and 15 (T + 15) minutes later. The plots show medians (line within box), mean (cross inside the box), 25th and 75th percentiles (boxes), min and max values (whiskers) and individual values (points). The numbers mean the name of the pigs. Asterisks indicate significant post-hoc difference (Dunn’s multiple comparisons test): * *P* < 0.05

sAA activity (Fig. 1a) showed a significant change between the times (*χ*^*2*^ = 12.76, df = 5, *P* = 0.026), but the multiple comparisons test did not show any significant increase at any time (*P* > 0.08 for all times compared). sAA concentration (Fig. 1b) did not show significant changes along the times (*χ*^*2*^ = 7.33, df = 5, *P* = 0.197). Salivary cortisol significantly changed between the times (*χ*^*2*^ = 13.71, df = 5, *P* = 0.033), with an increase (*P* = 0.045) at T + 0B compared to TB (Fig. 1c). There was a significant correlation between sAA concentration and salivary cortisol (*n* = 36, *r* = 0.48, 95% confidence interval [CI] 0.18–0.71, *P* = 0.003). However, no correlation was observed between sAA activity and sAA concentration (*n* = 36, *r* = 0.16, 95% CI − 0.18-0.47, *P* = 0.338), and between sAA activity and salivary cortisol (*n* = 36, *r* = 0.14, 95% CI − 0.21-0.45, *P* = 0.418). Recurrent high-pitched vocalizations were observed in the six pigs during the restraint period.

**Fig. 2:**
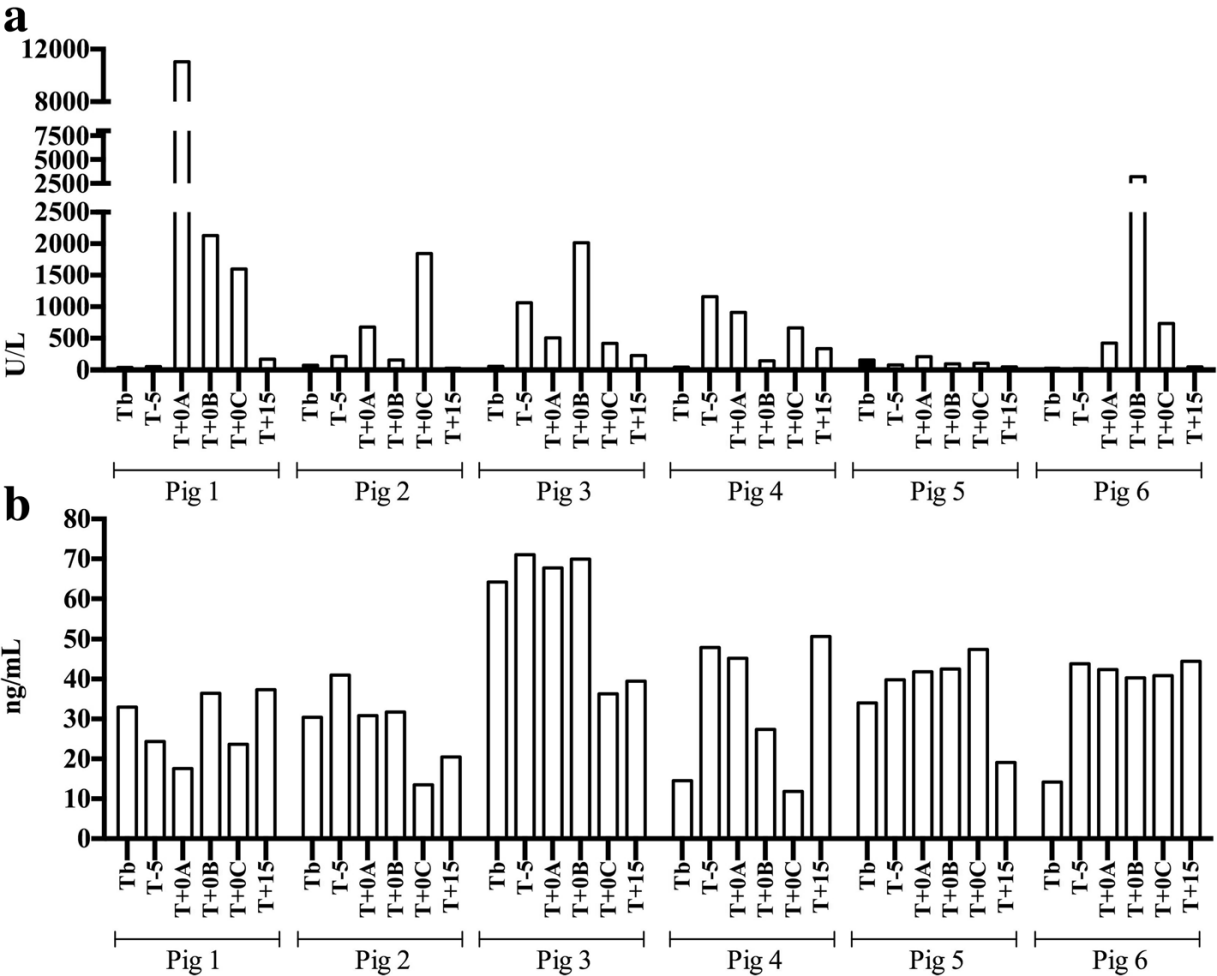
Individual levels of salivary alpha-amylase (sAA) activity (**a**) and concentration (**b**) obtained from six pigs before and after nasal snare immobilization for 1 min*.* The times were the day before at basal time (Tb), 5 min before (T-5), the firsts 30 s while the nasal snare immobilization was being performed (T + 0A), the seconds 30 s while the nasal snare immobilization was being performed (T + 0B), just after the immobilization (T + 0C) and 15 (T + 15) minutes later. Bars show the total value from each individual at each time

When sAA activity values were individually evaluated (Fig. 2a), the differences of sAA activity between the times had an average value higher than 1000 U/L in pigs 1 (4166.4 U/L) and 6 (1225.5 U/L); higher than 500 U/L in pigs 2 (743.6 U/L), 3 (826.2 U/L) and 4 (548.1 U/L); and lower than 100 U/L in pig 5 (71.1 U/L). Peaks of activity were observed in pig 1 at T + 0A, in pig 2 at T + 0C and in pigs 3 and 6 at T + 0B. In pig 4, the peak was observed at T-5.

When sAA concentration was individually evaluated between times (Fig. 2b), a decrease was observed at T + 0C in all pigs, with the exception of the pig 5 and 6. The inter-individual mean variability (Table 1) of the sAA activity (109.2% ± 42.2) was higher (2.5-fold and 5.1-fold, respectively) than in sAA concentration (42.7% ± 9.5) and salivary cortisol (21.2% ± 9.3).

**Table 1:** Inter-individual variability (%) of salivary alpha-amylase (sAA) measured by enzymatic activity (U/L) and concentration (ng/mL), and of salivary cortisol concentration (μg/dL) after the experimental nasal snare immobilization for 1 min in 6 pigs

sAA interindividual variability (%)			
	sAA		salivary cortisol
	U/L	ng/mL	μg/dL
Tb	75.3	57.51	22.5
T-5	123.6	34.09	14.42
T + 0A	187.0	40.64	18.61
T + 0B	103.5	36.33	8.77
T + 0C	76.2	51.16	34.2
T + 15	89.4	36.43	28.6
Mean	109.2	42.7	21.2
SD^a^	42.2	9.5	9.3

^a^*SD* standard deviation

### Changes in isoforms patterns

**Fig. 3:**
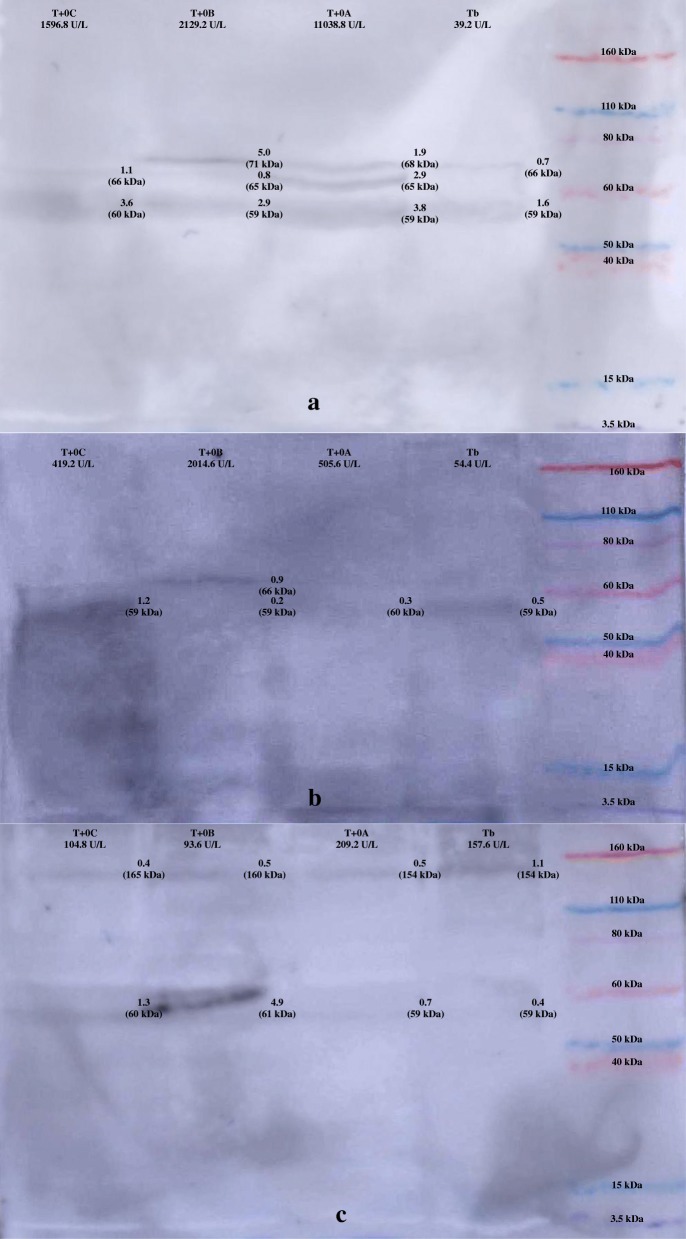
Western blot from pig saliva in an experimental nasal snare immobilization. Saliva samples from Pig 1 (**a**), Pig 3 (**b**) and Pig 5 (**c**) with one ng per well of salivary alpha-amylase (sAA) at the day before at basal time (Tb), the firsts 30 s while the nasal snare immobilization was being performed (T + 0A), the seconds 30 s while the nasal snare immobilization was being performed (T + 0B) and just after the immobilization (T + 0C). Molecular weight markers (Novex Sharp Pre-Stained, Invitrogen, Carlsbad, California). Arrows mark positive bands in pig’s saliva, and numbers above indicate the average intensity (× 10^3^)

WB results are presented in Fig. 3. Pigs saliva showed a band of 59–60 kDa in all times. On the other hand, pig 5 showed a band with 154–160 kDa along the different times. Additionally, a band at 65–66 kDa was observed in pigs 1 and 3 in the stress times only when sAA activity values were higher than 1000 U/L. The bands with 59–60 kDa and 154–160 kDa were also marked in the commercial human purified sAA (Additional file 1).

## Discussion

In this paper, changes in sAA activity, concentration, and its possible isoforms were evaluated after an experimental induced stress in pigs. The increase of salivary cortisol, considered as a biomarker of stress [7], that appeared in all pigs used in our study after the restraint, would indicate that our experimental procedure was able to generate a stress in the animals. In addition, the high-pitched vocalizations emitted by the six pigs during the restrain period could indicate a negative emotional state [5]. sAA activity showed peaks of increase in four of the six pigs after the stressor at different times and with different intensity, however sAA concentration decreased in many animals after the stimulus. A reason for this different behavior could be the fact that activity and concentration are associated to different possible isoforms of sAA.

For the analysis of sAA isoforms, serial samples from individuals representing different sAA response (one with a major sAA response, one with a moderate sAA response and one with a minor response) were analysed. According to the WB’s results, different sAA isoforms appeared depending of the intensity of the sAA response. In humans it has been demonstrated that these sAA isoforms can change after sAA stimulation [22] and changes observed in these isoforms could influence the concentration and activity of the enzyme. However, as well as occurs with activity, there was a high inter-individual variability in the response of the different isoforms. The bands with 59–60 kDa and 154–160 kDa observed in the WB from porcine saliva had the same molecular weight than bands marked in the commercial purified sAA from humans (Additional file 1). In addition, the band with 59–60 kDa could correspond to the glycosylated isoform described in humans [21]. So it could be postulated that the changes observed in the bands in the WB study could be due to different amylase isoforms. This could open a new line for the evaluation of possible selected isoforms of sAA as potential biomarkers of stress, since for example the isoform with a molecular weight of 65–66 kDa could be related with major response in sAA activity after an stress, since appeared in pigs showing the higher sAA activity response to a stressful stimulus, whereas the isoform of 154–160 kDa could be related with minor response in sAA activity after a stress condition.

A lack of correlation between sAA activity and concentration was observed in our study. Divergences between activity and concentration values have been described for other enzymes such as Paraoxonase-1 (PON1) in human serum [31]. In the sAA case, it could be postulated that post-translational changes [21] could modify the enzyme’s activity, although the concentration release does not show significant changes. In addition, we found no correlation between sAA activity and salivary cortisol. This is in line with previous reports previously described [15, 32] and could be due to the possible activation of the different related stress axis [33, 34] having a different time of response, with the sAA release being faster than the cortisol. Finally, the positive correlation between sAA concentration and salivary cortisol detected in our study should be taken with caution since *r* values below 0.50 are considered as weak [35], and both did not show a similar tendency along the times.

In this study, sAA activity showed a high inter-individual variability as it has been previously reported in pigs subjected to an acute stress model [15]. This high inter-individual variability was also observed in sAA concentration and it could be the cause for not obtaining significant differences in both sAA activity and concentration after the stress model. Biomarkers assessing of stress usually show inter-individual variability [8, 13, 36], probably due to the complexity of stress reaction [37] and because the stress response varies according to idiosyncrasy of the individual [38]. However, this inter-individual variability was higher in sAA activity, being in agreement with previous reports [10, 13, 20]. The higher inter-individual variability in sAA activity could be due to the fast-reacting biological response of the enzyme, and there is a large array of factors with potential to stimulate or inhibit amylase activity [13], that could be a potential limitation for its application as a stress biomarker.

It is important to point out that this is an exploratory study and the results observed should be taken as preliminary ones due to the low sample size. Further studies should be performed in large populations and with other different stress conditions in order to corroborate our findings. In addition, it would be of interest the development of studies to clarify the high inter-individual variability in sAA behavior after a stress induction that we found in our experimental conditions.

## Conclusion

This study reveals that sAA activity and concentration behave in a different way in pigs after an experimental acute stress stimulus, showing a high inter-individual variation in their response. In addition, it reports the presence of sAA isoforms in pig saliva that show a different response after a stress. Although these results should be taken as preliminary ones due to the pilot nature of this study with a low sample size, it could open a new line for the evaluation of possible selected isoforms of sAA as potential biomarkers of stress.

## Additional file

Additional file 1: Western blot from purified human salivary alpha-amylase (sAA, ab 77875, Abcam, Cambrigde, UK). Molecular weight markers (Novex Sharp Pre-Stained, Invitrogen, Carlsbad, California). (PDF 16 kb)

## Abbreviations

CI: Confidence interval
CP: Crude protein
CV: Coefficient of variation
HPA: Hypothalamic-pituitary-adrenocortical
HRP: Horseradish peroxidase
ME: Metabolizable energy
PON1: Paraoxonase-1
sAA: Salivary apha-amylase
SD: Standard deviation
SDS-PAGE: One-dimensional sodium dodecyl sulphate polyacrylamide gel electrophoresis
SNS: Sympathetic nervous system
TR-IFMA: Time-resolved immunofluorometric assay
WB: Western blot
