# Supplementary figures and images for: Changes in alpha-amylase activity, concentration and isoforms in pigs after an experimental acute stress model: an exploratory study
Source: BMC Vet Res. 2018 Aug 29;14:256. doi: 10.1186/s12917-018-1581-2 (PMC6116453; doi:10.1186/s12917-018-1581-2)

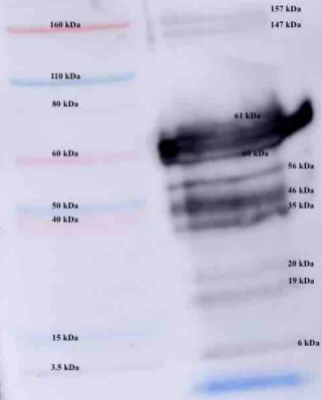

Supplement: Supplementary file 1 — Western blot from purified human salivary alpha-amylase (sAA, ab 77875, Abcam, Cambrigde, UK). Molecular weight markers (Novex Sharp Pre-Stained, Invitrogen, Carlsbad, California). (PDF 16 kb) [file 12917_2018_1581_MOESM1_ESM.pdf]
